# The Effect of Bilingualism on Cue-Based vs. Memory-Based Task Switching in Older Adults

**DOI:** 10.3389/fnhum.2020.610548

**Published:** 2020-12-18

**Authors:** Jennifer A. Rieker, José Manuel Reales, Soledad Ballesteros

**Affiliations:** ^1^Studies on Aging and Neurodegenerative Diseases Research Group, Madrid, Spain; ^2^Department of Basic Psychology II, Facultad de Psicología, Universidad Nacional, Madrid, Spain; ^3^Department Methodology of Behavioral Sciences, Facultad de Psicología, Universidad Nacional de Educación a Distancia, Madrid, Spain

**Keywords:** aging, bilingualism, cued task switching, memory-based task switching, executive function

## Abstract

Findings suggest a positive impact of bilingualism on cognition, including the later onset of dementia. However, it is not clear to what extent these effects are influenced by variations in attentional control demands in response to specific task requirements. In this study, 20 bilingual and 20 monolingual older adults performed a task-switching task under explicit task-cuing vs. memory-based switching conditions. In the cued condition, task switches occurred in random order and a visual cue signaled the next task to be performed. In the memory-based condition, the task alternated after every second trial in a predictable sequence without presenting a cue. The performance of bilinguals did not vary across experimental conditions, whereas monolinguals experienced a pronounced increase in response latencies and error rates in the cued condition. Both groups produced similar switch costs (difference in performance on switch trials as opposed to repeating trials within the mixed-task block) and mixing costs (difference in performance on repeat trials of a mixed-task block as opposed to trials of a single-task block), but bilinguals produced them with lower response latencies. The cognitive benefits of bilingualism seem not to apply to executive functions *per se* but to affect specific cognitive processes that involve task-relevant context processing. The present results suggest that lifelong bilingualism could promote in older adults a flexible adjustment to environmental cues, but only with increased task demands. However, due to the small sample size, the results should be interpreted with caution.

## Introduction

Modern societies are characterized by population aging due to increased life expectancy and falling birth rates, with older adults making up a growing proportion of the population (Gavrilov and Heuveline, 2003). This demographic aging implies exponential growth in the number of people who will experience age-related declines in cognition, and in the incidence and prevalence of dementia, and entails an important economic impact for caregivers and public health systems (World Health Organization, 2012; Hurd et al., 2013). However, not all people respond similarly to a neuropathological burden. While cerebral changes result in significant cognitive declines in some older adults, others can compensate for these changes and maintain their normal cognitive functioning up to advanced age (Riley et al., 2002). This phenomenon is referred to as cognitive reserve (Barulli and Stern, 2013).

Cognitive reserve is defined as the interindividual variability in how tasks are processed, allowing some people to cope better than others with brain pathology and age-related brain changes (Stern, 2009). Several activities and other environmental factors have been identified as fostering cognitive reserve, such as higher educational and occupational achievements (Bennett et al., 2003), or engaging in cognitively stimulating leisure activities (Ferreira et al., 2015; Ballesteros et al., 2018). It has been suggested that bilingualism contributes to this reserve as well, as it has been shown that, on average, bilinguals are diagnosed with Alzheimer’s Disease approximately 4 years later than monolinguals (Bialystok et al., 2007; Craik et al., 2010; Woumans et al., 2015), although some large prospective studies could not replicate this effect (for a recent review see Van den Noort et al., 2019). The benefits of the cognitive reserve can also be observed in healthy aging. Normal aging is associated with neurobiological changes that produce progressive declines in different cognitive domains (Park and Reuter-Lorenz, 2009; Reuter-Lorenz and Park, 2014), and most older adults manage to compensate for these cerebral changes by recruiting additional brain areas, or by overrecruiting frontal areas (Davis et al., 2008; Osorio et al., 2010). It appears that healthy older bilinguals perform non-verbal executive tasks without having to over-activate frontal areas (Gold et al., 2013; Ansaldo et al., 2015; for a recent review see Zhang et al., 2020) suggesting that the simultaneous management of two languages might lead to better maintenance of cerebral functionality in advanced age.

Bilinguals constantly need to monitor and control two different language codes that share the same neural substrate (Crinion et al., 2006), and one language is produced by inhibiting the other (Runnqvist et al., 2012). This increased demand for cognitive control seems to lead on some occasions to superior performance in tasks that involve executive functions (EF; see Adesope et al., 2010; Bialystok et al., 2012). Studies with children (Carlson and Meltzoff, 2008; Kapa and Colombo, 2013; for a review see Barac et al., 2014) and older adults (Bialystok et al., 2004; Salvatierra and Rosselli, 2010; Goral et al., 2015) have reported a bilingual advantage in executive control. With younger adults, results are more mixed (for reviews of results in young adults vs. results with children and older adults, see Bialystok, 2017; Antoniou, 2019), and bilingual brain mechanisms might compensate for lower-level executive functioning, for example, in childhood when executive functions are still developing (Casey et al., 2000), or in late adulthood when age-related decline appears (Zelazo et al., 2004). Several studies have shown that the bilingual advantage increases with task difficulty (Bialystok, 2006; Costa et al., 2009; Hernández et al., 2013; Qu et al., 2015). However, other studies have failed to find evidence for a cognitive benefit of bilingualism (Paap and Greenberg, 2013; Antón et al., 2016; Scaltritti et al., 2017). Different factors have been proposed as contributing to the inconsistencies found in the literature, such as task impurities when assessing EF (Hartanto and Yang, 2020), as well as differences in study designs, assessment tasks, and insufficient assessment of other variables known to modulate cognition such as physical exercise and cognitive stimulation (Calvo et al., 2016). Recent meta-analyses (Lehtonen et al., 2018; Donnelly et al., 2019) conclude that the average effect size for a bilingual advantage is small and that it disappears when controlling for publication bias (Paap et al., 2020). However, growing evidence suggests that attentional advantages might be related to long-term dual-language management (Stocco et al., 2014). The amount of the second language (L2) immersion (time spent in the country where L2 is spoken) and the frequency of language switching are important modulating factors of the effects of bilingualism on cognition (Prior and Gollan, 2011; Pliatsikas et al., 2016; Pot et al., 2018).

Most of the studies that have investigated EF in bilinguals have focused on inhibitory control (Bialystok et al., 2004; Costa et al., 2009) and task switching (Costa et al., 2008; Prior and Gollan, 2011; for a review see Bialystok, 2017). The assumption that inhibition is part of the mechanism for bilingual effects on cognition is based on the inhibitory control model (Green, 1998). According to this model, a supervisory attention system is guided by top-down cues, leading to the inhibition of the non-target language so that language processing can adapt to the contextual requirements. Extensions of this model (Green and Abutalebi, 2013; Green and Wei, 2014) include the differential influences of cognitive control processes as a function of the type of interactional context for language use and distinguish between three different contexts: (1) single-language; (2) dual-language; and (3) dense code-switching. In a single-language context, bilinguals use only one language in the same situation. In dual-language and code-switching contexts, bilinguals switch between the two languages in the same situation, but in the case of code-switching, languages are freely mixed in single utterances. Hartanto and Yang (2020) found that bilinguals with greater exposure to a dual-language context displayed significantly better task-switching abilities, replicating their findings of a previous study (Hartanto and Yang, 2016). They also found that dense code-switching was related to better inhibitory control and goal maintenance (Hartanto and Yang, 2020), a result that contrasts with a nonsignificant result regarding the relationship between dense code-switching and inhibitory control in another recent study (Kałamała et al., 2020). It seems that within dual-language contexts, situations that require constant goal reconfiguration and top-down control in response to outside constraints are more likely to translate into a cognitive advantage than free and unrestrained language switches (Blanco-Elorrieta and Pylkkänen, 2018).

On the other hand, the interest in the relationship between bilingualism and task-switching stems from behavioral data that show similar dynamics when shifting between dominant and less dominant templates (Meuter and Allport, 1999; Runnqvist et al., 2012). Further support for the commonalities between attentional set-shifting and dual-language management comes from neuroimaging evidence that shows an overlap in brain networks involved in language selection and nonverbal task switching (Meuter and Allport, 1999; Abutalebi and Green, 2007; Luk et al., 2011; Runnqvist et al., 2012; Baene et al., 2015; Coderre et al., 2016).

Cognitive processing of mental set-shifting might also vary as a function of task requirements. The conditional routing model (Stocco et al., 2010, 2014) proposes that bilingualism improves the ability to flexibly reallocate attention in complex and non-habitual task requirements, whereas the management of more direct stimulus-response mappings is not influenced by bilingual language processing. An example could be the reorientation in response to unpredictable external cues vs. reorientation in response to rule changes that occur in a sequenced order. In both cases, working memory (WM) plays an important role. WM allows for simultaneously maintaining and processing information to guide goal-directed behavior (Baddeley and Hitch, 1994). In memory-based, as well as in cued task switches, task sets need to be monitored and retrieved from memory and assembled with the correct stimulus-response mapping. However, the activation process is different for memory-based and randomly cued task switches. In memory-based set-shifting, the activation is triggered endogenously by a goal-directed monitorization in WM. When cued task switches occur randomly, the demand for a set shift is unpredictable and cannot be controlled by internal monitoring. In this case, the task-set activation is stimulus-driven; that is, triggered by a task-relevant cue (Corbetta et al., 2008).

Task-switching paradigms typically consist of blocks of switch and repeat trials and blocks of non-switch trials where only single-task sets are performed. The difference in performance between switch and repeat trials is called “switch cost” and reflects task-set reconfiguration processes associated with changing task sets across trials (Monsell, 2003). The difference in performance between repeat trials in the switch block and trials in the single-task block is called “mixing cost.” This difference is thought to reflect the active maintenance of multiple task configurations in working memory and is more sensitive to age-related cognitive changes (Kray and Lindenberger, 2000).

Task-switching paradigms comprise different variants of switch tasks. In the cued-switching version, shifts are generally random, and a cue signals the task to be performed next. In alternating-run versions, shifts occur in a predictable sequence after every N-trial, with or without the appearance of a cue. If no cue accompanies the sequence, then set-shifting is “memory-based,” as switches are triggered endogenously by working memory. To our knowledge, to date, only four studies have investigated task-switching abilities in older adults and three of them found significant group differences. Gold et al. (2013) analyzed performance in memory-based switching with predictable task sequences and found that bilinguals showed lower switch costs than their monolingual counterparts, with overall better levels of behavioral performance. Using a cued task-switching paradigm, Houtzager et al. (2015) found that switch and mixing costs were lower in the bilingual group. de Bruin et al. (2015) compared active and nonactive older bilinguals and monolinguals. They found a significant difference in raw switch costs between active bilinguals and monolinguals, which disappeared when controlling for baseline performance. Soveri et al. (2011) also used a cued task-switching paradigm, but their within-group design did not include a monolingual control group. Although the participants were slightly younger than in the other two studies, a positive relation was found between lower mixing costs and frequent language switching.

The present study had two main goals. The first was to investigate the influence of explicitly cued vs. memory-based switching conditions on the set-shifting abilities of bilingual and monolingual older adults. Specifically, we were interested to find out whether bilingualism would influence mental flexibility *per se*, or if differences between monolinguals and bilinguals would be more prominent when task switches were externally triggered (aleatory rule changes in response to a cued) in comparison to task switches that were endogenously triggered (memory-based sequential changes).

Therefore, our experimental design included two conditions requiring different types of attentional control: first, a memory-based switching condition based on the alternating-runs paradigm in which the task alternates every N-trial; second, a cued switching condition based on an explicit task-cuing paradigm with randomly alternating tasks, each preceded by an instructive cue (Monsell et al., 2003). Memory-based task switching is predictable and controlled endogenously by working memory processes (Monchi et al., 2001), whereas cued task-switching requires a context-dependent reorientation of attention (Monchi et al., 2001; Baene et al., 2015). Given the similarity of explicitly cued task switching and context-related dual-language management, we expected bilinguals to produce lower switch costs than monolinguals when task-set reconfiguration had to be adjusted in response to unpredictable external cues, whereas there would be no difference between groups when set-shifting was memory-based and triggered endogenously.

The second goal of our study was to investigate whether bilingualism influences age-related decline in WM. A large body of research has provided evidence of a positive relationship between cognitive aging and mixing costs (i.e., the difference between repeat trials of a mixed task block and non-switch trials of a single-task block; Kray and Lindenberger, 2000; Reimers and Maylor, 2005; Wasylyshyn et al., 2011; Huff et al., 2015). Mixing costs reflect the active maintenance of multiple task configurations in working memory and could be expected to increase when task switches are memory-based. However, the aging effect on mixing costs seems to increase with increasing task complexity (Kray, 2006; Terry and Sliwinski, 2012). Task complexity increases when rule changes are unpredictable and dependent on external cues, as the reconfiguration process additionally requires the correct interpretation and implementation of the informative cue (Tornay and Milán, 2001). For this reason, we expected to find larger mixing costs in the cued-switching condition than in the memory-based condition and that mixing costs would be larger in monolingual older adults than in bilingual older adults.

## Materials and Methods

### Participants

Forty-two older adults were recruited through flyers and media postings, informative talks at strategic locations, and snowball sampling (referrals from participants). The inclusion criteria were a score of 26 or above on the Mini-Mental State Examination (MMSE; Folstein et al., 1975), a score of below 5 on the Yesavage Geriatric Depression Scale (Yesavage et al., 1983; Spanish adaptation by Martínez de la Iglesia et al., 2002), no current history of psychiatric or neurological pathology, and for the monolingual participants, no mastery of a foreign language above the A1 level of the *Common European Framework of Reference for Languages* (CEFR). One bilingual participant did not meet the inclusion criteria (score above 5 on the depression scale) and was excluded from further analysis. Data of one monolingual participant was not recorded due to technical problems. Thus, the final sample was composed of 20 monolingual native Spanish older adults (eight males, M_age_ = 72.65, SD = 6.38, range = 60–83 years) and 20 German-Spanish bilingual older adults (four males, M_age_ = 72.25, SD = 9.12, range = 60–95 years). Table 1 summarizes the demographics and screening test scores for monolinguals and bilinguals. *T*-tests showed no significant differences between the two groups (all *p*s > 0.05) for all of these measures. Growing evidence suggests that the amount of the second language (L2) immersion (time spent in the country where L2 is spoken) and the frequency of language switching are important modulating factors of the effects of bilingualism on cognition (Prior and Gollan, 2011; Pliatsikas et al., 2016; Pot et al., 2018; Hartanto and Yang, 2020). Our bilingual sample was composed of highly balanced, late bilinguals who had been exposed to their L2-environment for more than 40 years on average. Fourteen bilinguals reported German as their first language (L1) and Spanish as their second language (L2), and six reported Spanish as their L1 and German as their L2. All participants were right-handed, had normal or corrected-to-normal vision and none reported color blindness.

**Table 1:** Mean values of socio-demographic background variables for monolinguals and bilinguals.

	Monolinguals	Bilinguals		
	(*n* = 20)	(*n* = 20)	*t* (*df*)	*p*
Men/women	8/12	4/16	*t*_(38)_ = −1.378	0.176
Age	72.25 (6.38)	72.65 (9.12)	*t*_(38)_ = −0.161	0.873
Education^1^	4.55 (2.06)	4.65 (1.42)	*t*_(38)_ = −0.178	0.859
MMSE^2^	28.85 (1.04)	29.3 (0.8)	*t*_(38)_ = −1.533	0.134
Depression^3^	1.2 (1.2)	0.7 (0.92)	*t*_(38)_ = −1.480	0.147

*^1^Level of educational attainment was defined as follows: 1 = Primary education, 2 = Lower secondary education, 3 = Post-secondary non-tertiary education, 4 = Upper secondary education, 5 = Short-cycle tertiary education, 6 = Bachelor’s or equivalent, 7 = Doctoral or equivalent. ^2^Mini-Mental State Examination (Folstein et al., 1975). ^3^Short Form of the Geriatric Depression Scale (GDS; Yesavage et al., 1983). SDs are shown in parentheses*.

Bilingualism was assessed with the validated Bilingual Language Profile questionnaire (BLP; Birdsong et al., 2012; see Table S1 for detailed information on the BLP). It has four components with a mean Cronbach’s alpha of 0.787 (Gertken et al., 2014): language history (e.g., “At what age did you start learning the following languages?” “How many years have you spent in a country/region where the following languages are spoken?”), language use (e.g., “In an average week, what percentage of the time do you use the following languages with friends?” “When you count, how often do you count in the following languages?”), language proficiency (e.g., “How well do you speak Spanish?” “How well do you read Spanish?”) and language attitudes (e.g., “I feel like myself when I speak Spanish,” “I identify with a Spanish-speaking culture”). For each component, two scores are computed (one for each language) and the difference between the two scores indicates the relative dominance of each language in that specific area. The scores for each component vary as follows: −120 to +120 for language history, −50 to +50 for usage, −24 to +24 for proficiency, and −24 to +24 for attitudes. The score of each component is multiplied by a weighting factor so that each component receives equal weighting (54.5) in the global language score. The difference between the total scores of the two languages constitutes the language dominance index, which ranges from −218 to +218. In the present study, we subtracted the German score from the Spanish score. A positive score indicated dominance in Spanish, and a negative score indicated dominance in German. A score of zero represents a balanced bilingualism. The linguistic background information for bilinguals is shown in Table 2. No statistically significant differences were found between monolinguals and bilinguals regarding the demographic background information.

**Table 2:** Mean values of socio-demographic background variables^1^ for monolinguals and bilinguals.

Spanish use (% week)	40 (21.82)
German use (% week)	60 (21.95)
Age of acquisition	19.9 (7.41)
BLP global score	−28.66 (61.93)
Language history	−12.3 (23.22)
Language use	−11.35 (23.93)
Language proficiency	−0.67 (9.49)
Language attitudes	−5.09 (15.77)

*^1^Bilingual Language Profile (BLP; Birdsong et al., 2012). Negative values indicate dominance in German. SDs are shown in parentheses*.

All participants gave their written informed consent. The study protocol was approved by the Institutional Review Board of the Universidad Nacional de Educación a Distancia (UNED) and the study was conducted following the ethical guidelines of the 1975 Declaration of Helsinki.

### Assessing Task Switching

**Figure 1:**
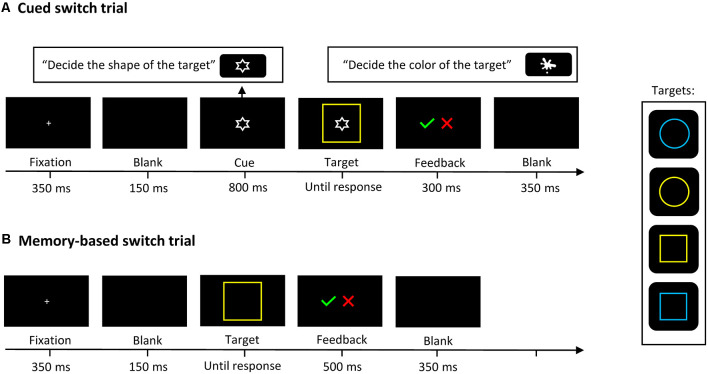
A schematic representation of the task-switching paradigm. In Cued switch trials **(A)**: an instructional cue indicated the next task to be performed. In Memory-based switch trials **(B)**: the task changed after every second trial without the appearance of a cue; that is, participants had to identify the shape of two consecutive stimuli and the color of the next two stimuli, and so forth. In single-task trials (not figured), participants only had to identify the color or the shape of the target.

The experimental task was adapted from Rubin and Meiran (2005) and contained three conditions: (1) in the single-task condition only one task had to be performed at a time; (2) in the cued-switching condition two tasks alternated in random order and a cue signaled the task to be performed next; and (3) in the memory-based switching condition two tasks alternated after every second trial without the appearance of a cue. It involved two bivalent target stimuli with two possible shapes (circle or square, both 60 × 60 mm) and in one of two possible colors (yellow or blue), presented in the center of the screen on a black background. In the cued-switching condition, a visual cue signaled the next task to be performed: a white splotch (18.8 mm) indicated that participants would have to identify the color of the target stimulus, and the white outline of a star (18.8 mm) that they would have to identify its shape. Although the cue was irrelevant in single-task blocks, it was presented in both single-task and cued-switching blocks to minimize differences between the conditions. In the memory-based switching condition, to help participants keep track of the correct trial sequence in the event of an error, two cues appeared on the screen (the same pictorial cues as in the cued-switching block), one indicating the correct condition of the just-completed trial, and one signaling the following trial condition. For a schematic representation of the task-switching paradigm, see Figure 1.

Each experimental run comprised eight blocks of trials. The first two blocks (23 trials each) were single-task blocks, one for shape and one for color. The third block was a cued-switching block with 46 trials (23 switch trials and 23 repeat trials), presented in a semi-random order with a maximum of three consecutive trials of the same condition and a maximum of two trials in which the condition and response mapping were identical to the preceding trial. The fourth block was a memory-based switching block, composed of 23 switch trials and 23 repeat trials. The following four blocks were a repetition of the previous trial blocks but in reverse order, starting with the memory-based switching block, followed by the cued-switching block, and ending with the single-task blocks. Altogether, the experiment contained 46 switch trials and 46 repeat trials in the cued condition, 46 switch trials and 46 repeat trials in the memory condition, and 92 non-switch trials (46 for color and 46 for shape) in the single-task condition, yielding a total of 276 trials per run.

### Procedure

Participants were tested individually in a single session. The experimental session lasted about 90 min. Stimuli were displayed on a laptop computer with a 15.6-inch monitor and a refresh rate of 60 Hz. Experimental scripts were designed, and data collection was managed with E-Prime 2.0 (Psychology Software Tools Inc., Pittsburg, PA, USA) experimental software. Participants were comfortably seated approximately 60 cm from the monitor. Non-switch trials and cued switch trials started with the presentation of the fixation point in the center of the screen for 350 ms, followed by a 150 ms blank screen. Then the instructional task cue appeared, and after 800 ms the target stimulus surrounded the cue and both stimuli remained on the screen until a response was given, or for a maximum of 10 s. Auditory feedback was presented for 300 ms (an incorrect response was followed by a low-frequency beep and a correct response by a high-frequency beep). The trial ended with a 350 ms blank screen. Memory-based switch trials also started with a 350 ms fixation point, followed by a 150 ms blank screen. Then the target stimulus appeared in the middle of the screen and remained until an answer was given or for 10 s. The auditory feedback was presented for 500 ms, and in the event of an incorrect response, two informative cues appeared on the screen simultaneously with the tone, indicating the correct response for the present task and the one that would follow. The trial ended with a 150 ms blank screen. At the beginning of each experimental block, written instructions for the upcoming task were displayed on the screen and remained until the space key was pressed. The response mapping was as follows: the *blue* response was assigned to the left index finger and the *yellow* response to the left middle finger. Similarly, the *square* response was assigned to the right index finger and the *circle* response to the right middle finger. The response keys for the color task were labeled with the appropriate colors, and the response keys for the shape task were labeled with the appropriate shape. Before beginning the actual task, participants performed 16 practice trials of each condition. Data from these practice trials were not included in the analyses.

### Data Analysis

RTs in color vs. shape judgments in single-task blocks did not differ significantly across participants (*t*_(39)_ = −0.072, *p* = 0.943, so we collapsed the data across the two conditions. For all reaction time (RT) analyses, only correct trials were included. Trials with response latencies below 200 ms and above 3,000 ms were excluded from the analysis. The RT-trimming procedure eliminated 2.28% and 2.93% of non-switch trials, 10.11% and 7.01% of repeat trials, and 12.55% and 8.26% of switch trials for monolinguals and bilinguals, respectively. In total, 7.19% of the trials were eliminated and were not included in the analysis. After data trimming, all distributions of response latencies showed acceptable levels of normality, homoscedasticity, and independence.

There were no negative associations between error rates and reaction times (RT) in any experimental condition, thus ruling out the possibility of a speed-accuracy trade-off. Error rates were analyzed using Mann–Whitney *U* tests. A significance level of *p* < 0.05 was adopted for all contrasts. Significance levels of multiple comparisons were Bonferroni-corrected to their number of comparisons. All the statistical analyses were conducted with SPSS v. 20.0 statistical software.

## Results

**Figure 2:**
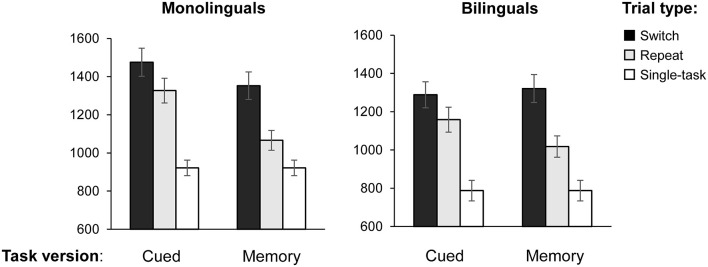
Mean reaction times (RTs) on the switch, repeat, and non-switch trials by task version (cued, memory-based, and single-task) for monolinguals and bilinguals. Error bars: ±1 SE.

Table 3 presents a summary of the response latencies, error rates, and composite switch and mixing costs per experimental and linguistic condition, and Figure 2 shows the response latencies by task version and trial type for monolinguals and bilinguals.

**Table 3:** Mean reaction time (RT) in milliseconds and error rates in the switch, repetition, and non-switch trials, and switch and mixing costs by experimental condition for monolinguals (*n* = 20) and bilinguals (*n* = 20).

Trial type	Task block	Monolinguals	Bilinguals
		**Response latencies in ms**	
Switch	Cued	1,475 (330)	1,288 (305)
	Memory	1,353 (322)	1,321 (328)
	Cued-Memory	123 (153)	−33 (158)
Repeat	Cued	1,327 (291)	1,158 (299)
	Memory	1,066 (234)	1,018 (250)
	Cued-Memory	260 (220)	140 (155)
Non-switch	Single task	921 (181)	787 (239)
		**Error rates in %**	
Switch	Cued	8.35 (5.5)	5.55 (4)
	Memory	6.25 (4)	4.05 (3)
Repeat	Cued	7.3 (5.5)	2.6 (2)
	Memory	5.05 (2)	3.5 (2)
Non-switch	Single task	1.15 (0)	1.45 (1)
		**Switch and mixing costs**	
Switch costs	Cued	148 (189)	130 (119)
	Memory	286 (179)	303 (155)
Mixing costs	Cued	406 (181)	371 (187)
	Memory	145 (173)	231 (151)

*SDs for RTs and Medians for error rates are shown in parentheses*.

### Switch Costs as a Function of Task Version

**Figure 3:**
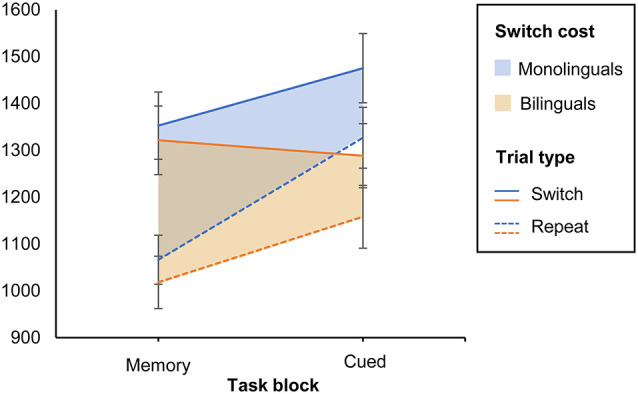
Switch costs by task version for monolinguals and bilinguals. The continuous lines indicate switch trials, and the discontinuous lines indicate repeat trials. The shadowed areas represent switch costs (i.e., the difference between both trial types). Error bars: ±1 SE.

Shifting attention to a new task requires more cognitive resources than the repetition of the same task. Switch costs are defined as the difference in performance on switch trials as opposed to repeat trials, within the mixed-task blocks. In our study, mixed-task blocks were either memory-based (task switches occurred after every second trial without the appearance of a cue) or cue-based (task switches occurred in random order and were triggered by a pictorial cue). To analyze the effect of both types of task settings on switch costs, we conducted a 2 (Group: monolinguals and bilinguals) × 2 (Task type: cued vs. memory-based) × 2 (Trial type: switch vs. repeat) mixed ANOVA on TR as the dependent variable, with Group as a between-subjects factor and Task and Trial type as within-subjects factors. The main effect of Task type was significant (*F*_(1,38)_ = 26.996, MSE = 22,250.218, *p* < 0.001, $\eta_{\text{p}}^{2}$ = 0.415, 1 − *β* = 0.999). Also, response latencies were larger on the switch than on repeat trials (*F*_(1,38)_ =101.077, MSE = 18,637.808, *p* < 0.001, $\eta_{\text{p}}^{2}$ = 0.727, 1 − *β* = 1, confirming that both task versions elicited switch costs for shifting attention. As indicated by a significant Task × Trial type interaction (*F*_(1,38)_ = 30.334, MSE = 7,045.376, *p* < 0.001, $\eta_{\text{p}}^{2}$ = 0.444, 1 − *β* = 1), response latencies increased from the memory-based to the cued version. This was especially the case in repeat trials, leading to smaller switch costs in the cued condition. We found a significant Group × Task interaction (*F*_(1,38)_ = 8.569, MSE = 22,250.218, *p* = 0.006, $\eta_{\text{p}}^{2}$ = 0.184, 1 − *β* = 0.814), suggesting that monolinguals and bilinguals adjusted in a different way to cued vs. memory-based task blocks. The magnitude of switch costs in both tasks was similar for monolinguals and bilinguals, as indicated by a non-significant main effect of Group (*p* = 0.219), and a non-significant three-way interaction Group × Trial × Task type (*p* = 0.383). To further investigate the significant Group × Task interaction, we performed Bonferroni corrected pairwise comparisons on the Group × Trial × Task interaction. Results revealed that, whereas monolinguals’ RTs were significantly larger on cued switch trials when compared to memory-based switch trials (mean difference = 123 ms, *p* = 0.001), the performance of bilinguals did not differ on switch trials of both task versions (mean difference = −33 ms, *p* = 0.35). See Figure 3. On repeat trials, both groups showed a similar pattern, with higher RTs in the cued than in the memory-based condition (mean difference = 260 ms, *p* = 0.01 and 140 ms, *p* = 0.01 for monolinguals and bilinguals, respectively).

An analysis of the error rates confirmed that the task repetition was more demanding for monolinguals than for bilinguals in a setting of unpredictable cued task switches. Monolinguals committed significantly more errors than bilinguals on cued repeat trials [monolinguals: 7.3% bilinguals: 2.6% (*U* = 109.5, *z* = −2.145, *p* = 0.012)]. The performance of the two groups did not differ in accuracy in the remaining factor levels, and error rates were overall lower in the memory-based condition (repeat trials: 4.28%, *p* = 0.665; switch trials: 5.1%, *p* = 0.455; switch trials: 5.1%, *p* = 0.455) than in the cue-based condition (switch trials: 6.95%, *p* = 0.494).

In sum, these results suggest that, when rule changes were triggered by external cues, bilinguals switched more efficiently between task sets across trials than monolinguals. These findings are congruent with the previously discussed literature in that bilinguals may allocate their cognitive resources in a more parsimonious way when task demands increase.

### Mixing Costs as a Function of Task Version

The repetition of a task rule in a context of set-shifting is always more effortful than performing the same task in a single-task context due to more complex task-set monitoring requirements (Monsell, 2003). This is what is indexed as “mixing costs” (i.e., the difference between repeat trials of a mixed task block and non-switch trials of a single-task block). To analyze the effect of single-task trials vs. repeat trials of both task versions, we conducted a 2 (Group: monolinguals and bilinguals) × 3 (Task type: single-task vs. memory-repeat trials vs. cued repeat trials) mixed ANOVA, with Group as a between-subjects factor and Trial type as a within-subjects factor. The main effect of Trial type was significant (*F*_(1,38)_ = 94.618, MSE = 16.082, *p* < 0.001, $\eta_{\text{p}}^{2}$ = 0.711, 1 − *β* = 1), indicating that the repetition of a trial in a mixed task block was overall more demanding than performing one task at a time. Neither the main effect of Group (*p* = 0.116), nor the Trial type × Group interaction resulted statistically significant (*p* = 0.094), suggesting that both groups produced similar mixing costs in both conditions. Bonferroni corrected pairwise comparison showed a trend for bilinguals being faster on single-task trials (*F*_(1,38)_ = 3.982, *p* < 0.052, $\eta_{\text{p}}^{2}$ = 0.095, 1 − *β* = 0.494) and on cued repeat trials (*F*_(1,38)_ = 3.271, *p* < 0.078, $\eta_{\text{p}}^{2}$ = 0.079, 1 − *β* = 0.422) whereas, as mentioned earlier, the performance on memory-based repeat trials was similar for both groups (*p* < 0.534).

To compare the magnitude of mixing costs as a function of task version, we ran an additional ANOVA, with Group as a between-subjects factor and Mixing cost (memory-based vs. cued) as within-subjects factors. The main factor of Mixing cost was significant (*F*_(1,38)_ = 44.353, MSE = 18,066.958, *p* < 0.001, $\eta_{\text{p}}^{2}$ = 0.539, 1 − *β* = 1), confirming that Mixing costs were overall higher in the cued condition (406 ms and 371 ms) than in the memory-based condition (145 ms and 231 ms, for monolinguals and bilinguals, respectively). A marginally significant Group × Mixing cost interaction (*F*_(1,38)_ = 4.028, MSE = 18,066.958, *p* = 0.052, $\eta_{\text{p}}^{2}$ = 0.096, 1 − *β* = 0.498) suggested that monolinguals experienced a larger increase in composite mixing costs from the cued to the memory-based task version (261 ms increase for monolinguals and 140 ms increase for bilinguals). Altogether, it seemed that both groups experienced an increase in the magnitude of mixing costs when task switches were unpredictable and externally cued and that this increase was slightly larger for monolinguals.

## Discussion

The results of the present study suggest that bilinguals shift their attention more efficiently than monolinguals when the task requirements mimic context-related dual-language management (i.e., aleatory and externally triggered task switches). The difference in response latencies between cued and memory-based switch trials was significantly larger in monolinguals than in bilinguals. The performance of bilinguals did not differ across task versions, whereas monolinguals experienced a pronounced increase in response latencies when set-shifting was unpredictable and triggered by an external cue. Task performance also differed in terms of accuracy, as monolinguals had a significantly higher error rate than bilinguals on cued repeat trials, suggesting that it was overall more effortful for them to shift attention under unpredictable task-switching conditions than it was for bilinguals. However, the magnitude of composite switch and mixing costs was similar for monolinguals and bilinguals, suggesting that composite scores might not sufficiently capture fine-grained differences in performance.

To compare task-switching abilities under different cognitive demands, in the present study we adapted a task-switching paradigm that contained both memory-based and cued task-switching blocks. This procedure served to tax slightly different underlying control mechanisms. The memory-based task-switching paradigm involves predictable sequences of rule changes and requires primarily the monitoring of information in working memory. By contrast, cued task-switching, like language-switching, additionally requires context-dependent attentional reorientation and increased cognitive control demands. Thus, we predicted that a bilingual advantage would only be found when set shifting was triggered externally. The results of this pilot study confirmed only partially this hypothesis. Monolinguals and bilinguals did not differ significantly in response latencies within each task version, but significant group differences were found in the dynamics between the two versions. The two groups performed almost identically in the memory-based switch task; hence this variable could be taken as baseline performance. Contrary to monolinguals, whose performance decreased, bilinguals maintained the same performance in the cued condition. Bilinguals had lower response latencies on cued switch trials and lower error rates on cued repeat trials, suggesting a bilingual advantage in the flexible adjustment to task-relevant context processing. These results are congruent with the existing literature regarding the similarity between cued task switching and linguistic code-switching (Christoffels et al., 2007; Prior and Gollan, 2011). Bilinguals might be more trained in efficiently interpreting contextual requirements to flexibly adjust their behavior. Previous research has shown that explicit cueing in a set of random-switching facilitates the task-set reconfiguration when enough time is given to prepare for the next trial (Tornay and Milán, 2001). Our experimental design included a cue-target interval of 800 ms, thus providing enough time for task preparation. Differences in efficient preparatory task-set activation are related primarily to individual differences in cognitive control, whereas age-related changes mainly appear to affect target response selection and task performance in general (Adrover-Roig and Barceló, 2010). In this line, our results suggest that cognitive aging affects the working-memory processes of monolinguals and bilinguals similarly, but that bilinguals might use contextual cues more efficiently and start the task-set reconfiguration earlier than monolinguals.

Our results also suggest that a long period of second-language immersion might parallel the cognitive benefits produced by an early age of acquisition. In our study, late bilinguals had been immersed in their second-language environment for more than 40 years on average and were highly balanced. However, dual-language exposure alone does not seem enough to modulate cognitive control. The balance in language use has been widely discussed as a core factor to explain the bilingual advantage (Verreyt et al., 2016; Yang et al., 2016; Hartanto and Yang, 2020). Even in balanced bilinguals, only high-frequency language switchers showed an advantage over monolinguals in tasks that measure cognitive flexibility (Barbu et al., 2020).

Long-time balanced dual-language immersion might lead to changes related to a more efficient reorientation to stimuli-driven task demands. As mentioned earlier, memory-based task switching requires more implication of WM sustained by an interaction of frontoparietal areas that are very sensitive to aging. Previous research has shown that, contrary to the so-called age-related posterior-anterior shift (PASA; Davis et al., 2008), this shift is reversed in some bilinguals to more subcortical/posterior regions during the performance of executive function tasks (Rodríguez-Pujadas et al., 2013; Grundy et al., 2017). Context-dependent reorientation (as in cued task-switching) relies on the interaction of frontostriatal loops with the special implication of the basal ganglia (Shulman et al., 2009; Van Schouwenburg et al., 2010). Several authors have proposed that at the initial stages of bilingualism, language control is mostly managed by prefrontal areas (Ullman, 2001; Stocco et al., 2014). Then, as dual-language management becomes more automatic, its neural processing shifts partly to subcortical areas (Lieberman, 2000; Tettamanti et al., 2005) as occurs in procedural knowledge (Packard and Knowlton, 2002). Bilinguals show expanded morphology in basal ganglia (Burgaleta et al., 2016). Damage to this brain area produces pathologic code-switching (Lieberman, 2000; Abutalebi and Green, 2008) similarly as it affects task-switching abilities in early Parkinson disease patients (Packard and Knowlton, 2002). Neuroimaging findings suggest that age-related changes in prefrontal areas affect bilinguals to a similar degree as monolinguals. However, bilinguals instead of overrecruiting those areas rely more on subcortical areas developed by life-long dual-language management. Our behavioral results fit with the current knowledge on bilingual neural processing and suggest that in older adults, processes that rely heavily on WM are affected similarly in monolinguals and bilinguals, but that bilingualism might improve processes that require a flexible reorientation to environmental cues.

Bilingualism is just one of the many components that might contribute to cognitive reserve. Numerous other factors and lifestyle habits can counteract their hypothetical benefits. Also, findings are heavily influenced by study design, and while retrospective studies tend to a protective effect of bilingualism on cognition, prospective studies often fail to find differences between monolinguals and bilinguals (Paap et al., 2016; Watson et al., 2016). The best alternative to investigate the effect of bilingualism on aging is to conduct powered randomized controlled trials that enable adequate control of baseline characteristics, psychological assessment, and experimental manipulations. To date, there are no results from such studies, but several promising study protocols, especially on the effect of foreign language learning in older adults, have recently been registered, and we can thus hope to obtain more insight into these important research questions in the near future.

## Limitations and Future Directions

A limitation of the present study is the small sample size. Possible differences between monolinguals and bilinguals, especially in composite switch and mixing costs, could be missed due to low statistical power. Small samples also increase the risk of type I errors, and the statistically significant interaction effect found in switch trials across conditions would need replication. However, the present study provides an innovative approach, contributing to the ongoing debate on the reliability of a bilingual advantage and prepares the ground for a larger-scale investigation, focusing not only on bilingual balance and language use but also on specific task characteristics.

## Data Availability Statement

The raw data supporting the conclusions of this article will be made available by the authors, without undue reservation.

## Ethics Statement

The studies involving human participants were reviewed and approved by Institutional Review Board of the Universidad Nacional de Educación a Distancia (UNED). The patients/participants provided their written informed consent to participate in this study.

## Author Contributions

JAR and SB: conceptualization and study design. JAR: enrolled the participants, collected, and analyzed the data. JAR and JMR: data analysis. All the authors: interpretation and final approval. JAR with support from SB: manuscript preparation. All authors contributed to the article and approved the submitted version.

## Conflict of Interest

The authors declare that the research was conducted in the absence of any commercial or financial relationships that could be construed as a potential conflict of interest.
